# Evidence for a prosurvival role of alpha‐7 nicotinic acetylcholine receptor in alternatively (M2)‐activated macrophages

**DOI:** 10.1002/phy2.189

**Published:** 2013-12-26

**Authors:** Robert H. Lee, Guillermo Vazquez

**Affiliations:** 1 Department of Physiology and Pharmacology, Center for Diabetes and Endocrine Research, University of Toledo College of Medicine, Health Science Campus, 3000 Arlington Av, Toledo, 43614, Ohio, USA

**Keywords:** Macrophage polarization, macrophage survival, *α*7 nicotinic acetylcholine receptor

## Abstract

Recent observations in endothelial cells and macrophages indicate that nicotinic acetylcholine receptors (nAChRs) are potential novel players in mechanisms linked to atherogenesis. In macrophages, *α*7nAChR mediates anti‐inflammatory actions and contributes to regulation of cholesterol flux and phagocytosis. Considering that macrophage apoptosis is a key process throughout all stages of atherosclerotic lesion development, in the present study, we examined for the first time the impact of *α*7nAChR expression and function in macrophage survival and apoptosis using in vitro polarized (M1 and M2) bone marrow‐derived macrophages (BMDMs) from wild‐type and *α*7nAChR knockout mice. Our findings show that stimulation of *α*7nAChR results in activation of the STAT3 prosurvival pathway and protection of macrophages from endoplasmic reticulum (ER) stress‐induced apoptosis. These actions are rather selective for M2 BMDMs and are associated to activation of the JAK2/STAT3 axis. Remarkably, these effects are completely lost in M2 macrophages lacking *α*7nAChR.

## Abstract (precis)

Macrophage apoptosis is a key process throughout all stages of inflammatory vascular disease. Our studies examine for the first time the impact of *α*7nAChR expression and function in macrophage survival and apoptosis using in vitro polarized (M1 and M2) bone marrow‐derived macrophages (BMDMs) from wild‐type and *α*7nAChR knockout mice. We show that stimulation of *α*7nAChR activates the STAT3 prosurvival pathway and protects macrophages from endoplasmic reticulum stress‐induced apoptosis, an effect rather selective for M2 macrophages and completely lost in M2 macrophages lacking *α*7nAChR.

## Introduction

Macrophages are now recognized to play a determinant role in the pathogenesis of the atherosclerotic lesion (Seimon and Tabas 2009; Tabas 2010). In the context of the cellular and molecular events that contribute to lesion development, the balance between survival and apoptosis of lesional macrophages and the timely clearance of apoptotic macrophages from the lesion site by resident phagocytes – efferocytosis – are critical in determining lesion cellularity and progression (Tabas 2010). Notably, a progressive deficiency in efferocytosis combined with an increased rate of macrophage apoptosis – mostly subsequent to endoplasmic reticulum (ER) stress – are key contributors to enlargement of the lesion necrotic core and plaque instability (Tabas 2009; Korns et al. 2011). Therefore, identifying and characterizing molecular mechanisms that control macrophage survival, apoptosis, and/or efferocytosis is mandatory in order to pinpoint potential targets that could be exploited to manipulate macrophage's function and fate during disease.

Nicotinic acetylcholine receptors (nAChRs) constitute a family of ligand‐gated nonselective cation channels of a pentameric structure (Nai et al. 2003; Barrantes et al. 2010). At least 16 different subunits have been identified in humans and mice (*α*1–*α*7, *α*9–10, *β*1–*β*4, *δ*, ε, *γ*), which can form a large number of homo and heteropentameric arrangements of functional channels. Besides the well‐characterized roles of nAChRs in neuromuscular junctions and cholinergic synapses in central and peripheral nervous systems, a significant amount of evidence has accumulated indicating important roles of nAChRs in nonneuronal tissues and organ systems, where they contribute to physiopathological processes. Indeed, over recent years, nAChRs have emerged as potential novel modulators of mechanisms linked to the pathogenesis of atherosclerosis, by virtue of their functions in a number of endothelial and macrophage ‐related processes. In the particular case of macrophages, a potential anti‐inflammatory role of *α*7nAChR was recently examined in peritoneal macrophages derived from *α*7nAChR‐deficient mice (Wilund et al. 2009). These in vitro studies indicated that *α*7nAChR may contribute to regulation of macrophage cholesterol metabolism and phagocytosis (Wilund et al. 2009). However, it is not evident from the available data whether the role of macrophage *α*7nAChR is truly relevant to atherosclerosis. In cells other than macrophages, such as neurons, lymphocytes, and coronary endothelial cells, stimulation of the *α*7nAChR results in activation of survival pathways and reduced apoptosis (De Rosa et al. 2009; Akaike et al. 2010; Smedlund et al. 2011). Thus, considering the influence of macrophage survival and apoptosis in atherogenesis, it is particularly intriguing whether the *α*7nAChR participates in the signaling associated to those events.

The present studies were aimed at examining the potential role of *α*7nAChR in macrophage survival and apoptosis using in vitro polarized (M1 and M2) bone marrow‐derived macrophages (BMDMs) from wild‐type and *α*7nAChR deficient mice.

Our findings indicate that selective activation of *α*7nAChR activates STAT3, a recognized prosurvival pathway in macrophages (Liu et al. 2003; Li et al. 2008), and protects these cells from ER stress‐induced apoptosis. Notably, this protective effect selectively benefits M2 BMDMs and is lost in M2 macrophages lacking *α*7nAChR. We discuss our findings in the context of the potential role of *α*7nAChR in macrophage function during atherogenesis.

## Material and Methods

### Experimental animals

All studies involving animals described in this work conform to the Guide for the Care and Use of Laboratory Animals published by the U.S. National Institutes of Health and have been approved by University of Toledo IACUC. C57BL/6 mice and the *α*7nAChR knockout mice were obtained from Jackson Labs (Jackson Labs, Bar Harbor, ME) and colonies were maintained in our animal facility. Euthanasia was performed by intraperitoneal injection of sodium pentobarbital (150 mg/kg) added to an anticoagulant (heparin, 10 U/mL).

### Preparation of bone marrow‐derived macrophages

Bone marrow‐derived macrophages were obtained as we described in Tano et al. (2011). Briefly, femurs and tibias were flushed with sterile RPMI (2% fetal bovine serum + 5 U/mL heparin + 1% penicillin/streptomycin) and cells were plated with L929‐conditioned medium for 7 days (37°C, 5% CO_2_ atmosphere); after that, cells were replated in 6‐well (immunoblotting) and 96‐well (terminal deoxynucleotidyl transferase dUTP nick end labeling (TUNEL)) plates for experiments. At this point, and before proceeding with the macrophage polarization protocol (see below), the macrophage phenotype of the cells was confirmed (>99%) by their cobblestone appearance, positive immunostaining with F4/80 antibody and Acetylated‐LDL uptake, as we previously described in Tano and Vazquez (2011). Please note that L929 cells (ATCC, CCl‐1, mouse fibroblastic cell line) were grown in RPMI + 10% fetal bovine serum + 1% penicillin/streptomycin during 7 days; the supernatant (cell‐free) was collected, filtered (0.22 *μ*m pore), and added to the macrophage differentiation media at a final concentration of 30%.

### Macrophage polarization into M1 and M2 phenotypes

Bone marrow‐derived macrophages (see Tano et al. 2011) were recovered with ice‐cold PBS, collected by centrifugation (380× g), and plated in complete medium (RPMI + 10% FBS + 1% penicillin/streptomycin) containing either 10 ng/mL interferon‐*γ* (IFN*γ*; M1 polarization) or 5 ng/mL IL‐4 (M2 polarization, predominantly M2a) for 24 h, as we described in Tano et al. (2013). After 24 h, macrophages were treated for experiments as noted. Polarization to the M1 or M2 phenotype was confirmed by qRT‐PCR with primers for markers of M1 (iNOS, inducible nitric oxide synthase; TNF*α*, tumor necrosis factor *α*) and M2 macrophages (ArgI, mannose receptor (MR)) as we described in Tano et al. (2013) (and see “Results” for details). IFN*γ* and IL‐4 were from Millipore (Billerica, MA).

### In vitro TUNEL assay

Apoptosis was assayed by using the in situ cell death detection kit, TMR red (Roche, Indianapolis, IN) as we described in Tano et al. (2012a).

### Cell lysis and immunoblotting

Essentially as we described in (Tano and Vazquez 2011; Tano et al. 2011). Briefly, following cell lysis, solubilized proteins were separated in 10% acrylamide gels, electrotransferred to PVDF membranes and immunoblotted with the indicated primary antibody. After incubation with appropriate HRP‐conjugated secondary antibodies, immunoreactive bands were visualized by ECL (Amersham, Pittsburgh, PA). Immunoblots examining changes in STAT3 phosphorylation were normalized against total STAT3; whereas the latter did not show significant variation over the experimental period of time (up to 60 min), control blots were also run in which total STAT3 levels were evaluated against the reference protein glyceraldehyde 3‐phosphate dehydrogenase (GAPDH), confirming that no variations occurred in total STAT3 over the duration of the experiments under any experimental condition. Primary antibodies used were: phospho‐AKT (Ser473, clone 587F11), total AKT, phospho‐p38 MAPK (Thr180/Tyr182, clone D3F9), total p38MAPK, phospho‐STAT3 (Tyr705, clone 3E2), total STAT3, phospho‐ERK1/2 (Thr202/Tyr204 of ERK1, Thr185/Tyr187 of ERK2), and total ERK1/2, and were all obtained from Cell Signaling (MA).

### Real‐time PCR (RT‐PCR)

Total RNA was prepared from BMDMs using PerfectPure RNA Tissue kit (5Prime, Gaithersburg, MD) according to manufacturer's instructions. cDNA was synthesized with random primers and reverse transcriptase (Applied Biosystems high‐capacity cDNA RT kit; Applied Biosystems, Grand Island, NY) using 1 *μ*g of total RNA. cDNA was evaluated with semiquantitative real‐time PCR (qRT‐PCR) using TrueAmp SYBR green qPCR supermix (Applied Biosystems). The relative amount of mRNA was calculated by comparison to the corresponding standards and normalized relative to GAPDH. Results are expressed as mean ± SEM relative to controls. Sequences of primers used are as follows: Arginase I (F: CAGAAGAATGGAAGAGTCAG; R: CAGATATGCAGGGAGTCACC), iNOS (F: TGCATGGACCAGTATAAGGCAAGC; R: GCTTCTGGTCGATGTCATGAGCAA), TNF*α* (F: CAGGCGGTGCCTATGTCTC; R: CGATCACCCCGAAGTTCAGTAG), MR (F: CTCTGTTCAGCTATTGGACGC; R: CGGAATTTCTGGGATTCAGCTTC), *α*7nAChR (F: AATTGGTGTGCATGGTTTCT; R: AGCCAATGTAGAGCAGGTTG), Bcl‐2 (F: ATGCCTTTGTGGAACTATATGGC; R: GGTATGCACCCAGAGTGATGC), GAPDH (F‐ AGGTCGGTGTGAACGGATTTG; R‐ TGTAGACCATGTAGTTGAGGTCA. Primerbank (Spandidos et al. 2010; Wang et al. 2012) identification numbers: TNF*α*: 133892368c1; MR: 224967061c1; Bcl‐2: 6753168a1; GAPDH: 6679937a1. Primers for Arginase I and iNOS were as in Khallou‐Laschet et al. (2010b). Primers for *α*7nAChR were ordered from Realtimeprimers.com (#VMPS‐1143). PCR for *α*7nAChR was run for 35 cycles. The specificity of all primers used in qRT‐PCR was evaluated from melting curve analysis.

### Statistical analysis

Values are shown as mean ± SEM and corresponding “*n*” indicated in figure legends or text. Comparison of mean values between groups was performed with a two‐tailed Student's *t* test. Statistical analysis was performed using Prism Graph Pad version 6 for Windows 2007 (Graph Pad Software, San Diego, CA). *P* values <0.05 were considered significant.

## Results

### *α*7nAChR deficiency does not affect macrophage polarization in vitro

**Figure 1.**
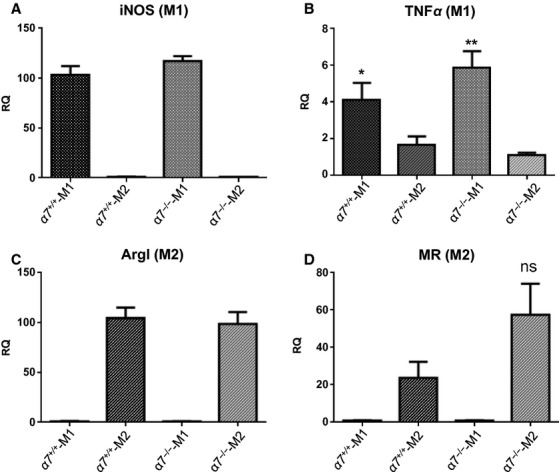
Bone marrow‐derived macrophages (BMDMs) obtained from wild‐type (α7^+/+^) or α7nAChR knockout (α7^−/−^) mice were polarized to M1 or M2 phenotypes and RNA and cDNA were prepared as described in Methods. Expression of specific markers for M1 (A, B) and M2 (C, D) macrophage phenotypes were measured by qRT‐PCR. iNOS, inducible nitric oxide synthase; TNFα, tumor necrosis factor α; ArgI, arginase I; MR, mannose receptor. Graphs represent data (mean ± SEM) of four independent experiments. Gene expression was normalized with GAPDH as an endogenous control. In (B) *P = 0.04 for the difference between α7^+/+^‐M1 and α7^+/+^‐M2; **P = 0.002 for the difference between α7^−/−^‐M1 and α7^−/−^‐M2. There was no statistically significant difference between α7^+/+^‐M1 and α7^−/−^‐M1 (*P* = 0.243). In (D) ns: not statistically significant for the difference between α7^−/−^‐M2 and α7^+/+^‐M2. Sequences for primers are provided in Materials and Methods.

Available evidence supports the notion that in vivo and in an inflammatory setting such as that accompanying atherosclerotic lesion development, macrophages take on a wide variety of phenotypes, with classically activated M1 and alternatively activated M2 macrophages being relatively well characterized (Mantovani et al. 2009). Before examining the impact of macrophage deficiency of *α*7nAChR on survival and apoptosis, we sought to evaluate if *α*7nAChR expression influenced macrophage polarization. We generated BMDMs from wild‐type and *α*7nAChR^−/−^ mice (from now on *α*7^+/+^ and *α*7^−/−^, respectively, both on C57BL/6 background) and induced polarization by treatment with either IFN*γ* (10 ng/mL, 24 h; M1 phenotype) or interleukin‐4 (IL‐4, 5 ng/mL; M2 phenotype; see Materials and Methods for details). To confirm polarization to the desired phenotype, we examined expression of specific markers of M1 and M2 differentiation by semiquantitative real‐time PCR (qRT‐PCR; Martinez et al. 2008; Khallou‐Laschet et al. 2010b); the M1 phenotype was assessed by examining expression levels of iNOS and TNF*α*, which are typically upregulated by IFN*γ* in M1 macrophages only, whereas arginase I (ArgI) and MR (CD206) were evaluated as M2 markers that respond to IL‐4. As expected, IFN*γ* treatment resulted in prominent upregulation of iNOS and TNF*α* in M1 macrophages from *α*7^+/+^ and *α*7^−/−^ mice but not in M2 cells, while M2 macrophages had high levels of ArgI and MR (Fig. 1). Importantly, when comparing expression levels of M1‐ or M2‐specific markers between *α*7^+/+^ and *α*7^−/−^ macrophages, no significant differences were observed.

### Expression of *α*7nAChR in polarized macrophages

**Figure 2.**
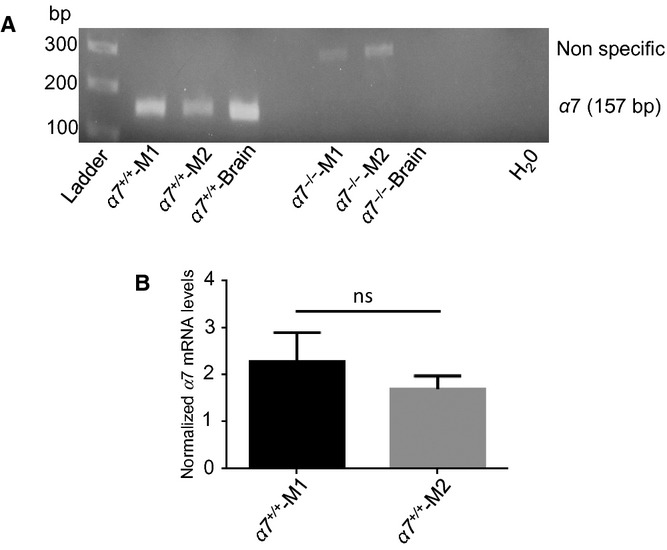
Bone marrow‐derived macrophages (BMDMs) obtained from wild‐type (α7^+/+^) or α7nAChR knockout (α7^−/−^) mice were polarized to M1 or M2 phenotypes, and RNA and cDNA were prepared as described in Methods. (A) Expression of α7 subunit (157 bp) of the α7nAChR was examined by PCR. Brain cDNA was used as a positive control for α7 subunit expression. (B) qRT‐PCR on cDNA from M1 and M2 BMDMs from α7^+/+^ mice. Results are shown as mRNA levels for α7nAChR normalized against GAPDH. Bars represent data (mean ± SEM) of five independent experiments, each performed in triplicates. ns, not statistically different. Sequences for primers used in “A” and “B” are provided in Materials and Methods.

The *α*7nAChR has been shown to be expressed in both human monocyte‐derived macrophages (Wang et al. 2003) and murine peritoneal and alveolar macrophages (De Simone et al. 2005; Kawashima et al. 2007; Su et al. 2007). To examine the expression of *α*7nAChR in polarized BMDMs, we collected total RNA from *α*7^+/+^ and *α*7^−/−^ M1‐ and M2‐polarized BMDMs to perform PCR for *α*7nAChR. PCR using primers specific for the *α*7nAChR subunit showed amplicons of the expected size in both *α*7^+/+^ M1 and M2 macrophages, which were absent in the *α*7^−/−^ BMDMs (Fig. 2A). The identity of the 157 bp amplicon was confirmed by the direct sequencing of the excised band (not shown). Furthermore, we performed qRT‐PCR to examine relative expression levels between the two macrophage populations. There was no significant difference in *α*7nAChR transcript levels between *α*7^+/+^ M1 and M2 macrophages (Fig. 2B).

### Nicotinic receptor stimulation activates survival signaling pathways in polarized BMDMs

**Figure 3.**
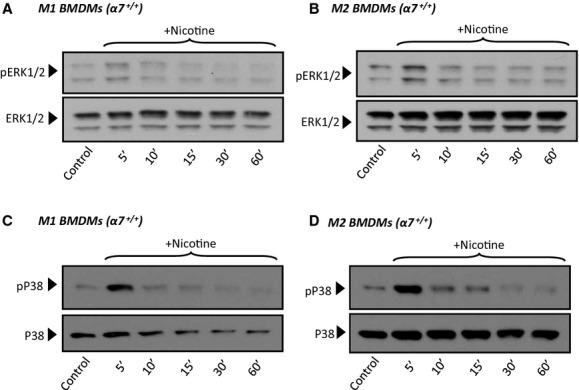
Bone marrow‐derived macrophages (BMDMs) obtained from wild‐type mice were polarized to M1 (A, C) or M2 (B, D) phenotypes, treated with nicotine (10 μmol/L) for the indicated times and then processed for immunodetection of A, B) phospho‐ERK1/2 (Thr202/Tyr204 of ERK1, Thr185/Tyr187 of ERK2; 42/44 kDa) or (C, D) phospho‐P38 MAPK (Thr180/Tyr182) in whole cell lysates. Membranes were reprobed for total ERK1/2 or total P38MAPK to control for protein loading. Blots are representative of three independent experiments.

**Figure 4.**
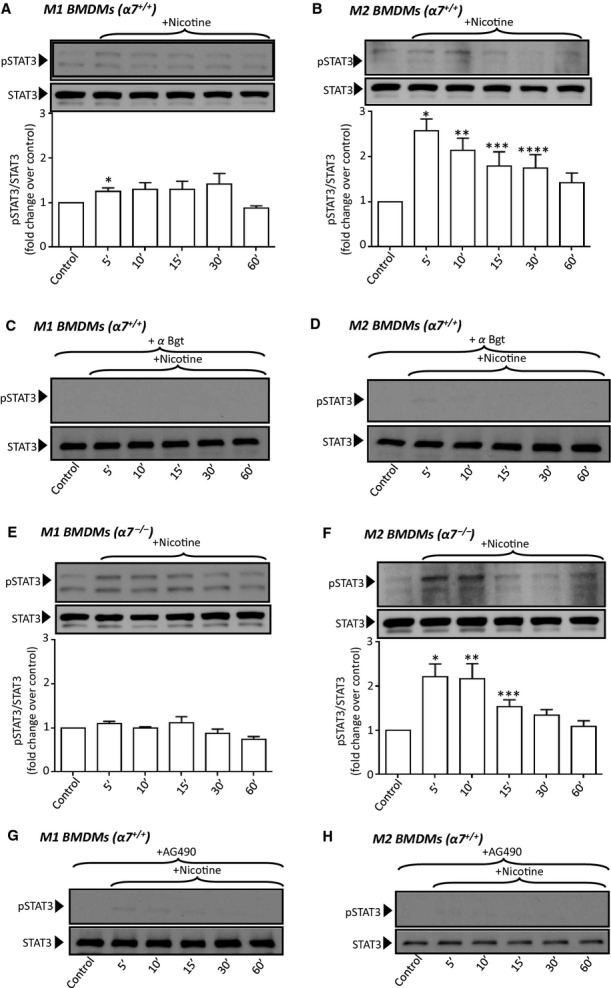
Bone marrow‐derived macrophages (BMDMs) obtained from wild‐type (α7^+/+^) mice were polarized to M1 (A, C, G) or M2 (B, D, H) phenotypes, treated with nicotine (10 μmol/L) for the indicated times in the absence (A, B) or presence (C, D) of α‐bungarotoxin (“αBgt”, 100 nmol/L, 15 min preincubation for all conditions, as indicated) or in the presence (G, H) of AG490 (10 μmol/L, 15 min preincubation for all conditions, as indicated). Alternatively, BMDMs from α7nAChR knockout (α7^−/−^) mice were polarized to M1 (E) or M2 (F) types and treated with nicotine (10 μmol/L) for the indicated times. In all instances, macrophages were processed for immunodetection of phospho‐STAT3 (Tyr705) in whole cell lysates. Membranes were reprobed for total STAT3 to control for protein loading. In A, B, E, and F, bar graphs below the blots show averaged normalized densitometry values from three independent experiments. In A, *P = 0.024; in B, *P = 0.0001, **P = 0.002, ***P = 0.032, ****P = 0.03; in F, *P = 0.001, **P = 0.006, ***P = 0.02.

We and others have previously shown that stimulation of *α*7nAChRs in cells other than macrophages results in activation of survival signaling pathways including ERK1/2 MAPK, p38MAPK, AKT, and STAT3 (Gubbins et al. 2010; Smedlund et al. 2011). Using polarized *α*7^+/+^ and *α*7^−/−^ BMDMs, we examined if activation of survival signaling occurs following nAChR stimulation. When M1 and M2 *α*7^+/+^ macrophages were treated with nicotine (10 *μ*mol/L), a nonspecific agonist for all nAChRs, we observed a rapid and transient phosphorylation of ERK1/2 and p38MAPK, as indicated by the extent of phosphorylation of Thr202/Tyr204 (ERK1), Thr185/Tyr187 (ERK2), and Thr180/Tyr182 (p38MAPK), respectively, without significant changes in total levels of ERK1/2 and p38MAPK (Fig. 3). Under these conditions, the phosphorylation status of AKT was not significantly altered (not shown). Similarly, nicotine treatment also resulted in rapid phosphorylation of STAT3 (Tyr705; Fig. 4). When compared with the corresponding basal levels, nicotine‐induced STAT3 phosphorylation was more robust and sustained in M2 than in M1 BMDMs (2.58 ± 0.25 vs. 1.25 ± 0.08, fold induction over control for M2 and M1, respectively, *P* = 0.003, *n* = 4). Interestingly, STAT3 phosphorylation was reduced (~40% at 5–10 min) by *α*‐bungarotoxin (100 nmol//L, Fig. 4C and D), an antagonist of *α*1, *α*7, and *α*9nAChRs (Bray et al. 2005; Albuquerque et al. 2009); of importance; by means of RT‐PCR, we did not detect expression of *α*1 or *α*9nAChRs in BMDMs (data not shown; and see Discussion). Also, the magnitude of STAT3 phosphorylation was reduced and less sustained in *α*7^−/−^ BMDMs (Fig. 4E and F). Altogether, these results suggest that nicotinic stimulation of STAT3 phosphorylation was contributed, to a significant extent, by *α*7nAChR. Nicotine‐dependent regulation of ERK1/2 and p38MAPK was not affected by *α*‐bungarotoxin or *α*7nAChR deficiency (not shown), suggesting that in polarized BMDMs nicotinic activation of these pathways may involve receptors other than *α*7nAChR (and see Discussion). Previous studies have shown that phosphorylation of STAT3 subsequent to nAChR stimulation is regulated by the Janus kinase 2 (JAK2; de Jonge et al. 2005). To examine if this was the case in polarized BMDMs, we pretreated M1 and M2 macrophages with the JAK2 inhibitor AG‐490 prior to nicotine treatment. Inhibition of JAK2 abrogated nicotine‐induced STAT3 phosphorylation suggesting that nicotinic receptor stimulation activates STAT3 in a JAK2‐dependent manner (Fig. 4G and H).

**Figure 5.**
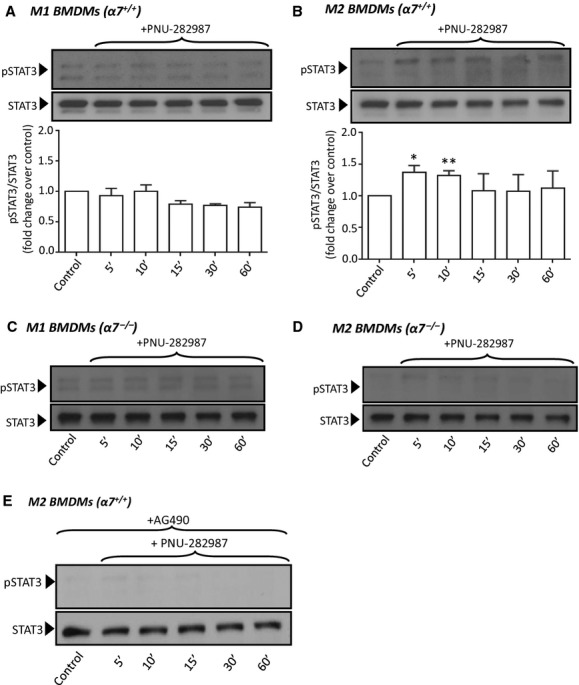
Bone marrow‐derived macrophages (BMDMs) obtained from wild‐type (α7^+/+^, A, B and E) or α7nAChR knockout (α7^−/−^, C and D) mice were polarized to M1 (A, C) or M2 (B, D, E) phenotypes, treated with PNU‐282987 (1 μmol/L) for the indicated times and then processed for immunodetection of phospho‐STAT3 (Tyr705) in whole cell lysates. In “E”, cells were incubated with AG490 (10 μmol/L, 15 min preincubation for all conditions, as indicated) before PNU‐282987 treatments. Membranes were reprobed for total STAT3 to control for protein loading. In A and B, bar graphs below the blots show averaged normalized densitometry values from three independent experiments. *P = 0.023, **P = 0.016.

To better define the role of *α*7nAChR in macrophage survival in the following experiments, we focused on the impact of *α*7nAChR activation or expression on the STAT3 prosurvival pathway. To specifically examine the involvement of *α*7nAChR, we treated macrophages with PNU‐282987, a selective *α*7nAChR agonist of the quinuclidine benzamide class (Bodnar et al. 2005). Treatment of *α*7^+/+^‐ or *α*7^−/−^‐M1 macrophages with PNU‐282987 did not show significant changes in the phosphorylation status of STAT3 (Fig. 5A and C). Contrarily, PNU‐282987 induced a rapid and transient phosphorylation of STAT3 in *α*7^+/+^‐M2 macrophages (~40–50% increase at 5–10 min) and this effect was markedly reduced in M2 cells lacking *α*7nAChR expression (Fig. 5B and D). PNU‐282987 activation of STAT3 in *α*7^+/+^‐M2 was completely abrogated by pretreatment with the JAK2 inhibitor AG‐490 (Fig. 5E), again suggesting operation of a *α*7nAChR/JAK2/STAT3 axis in M2 macrophages.

### Stimulation of *α*7nAChR protects M2 macrophages from ER stress‐induced apoptosis

**Figure 6.**
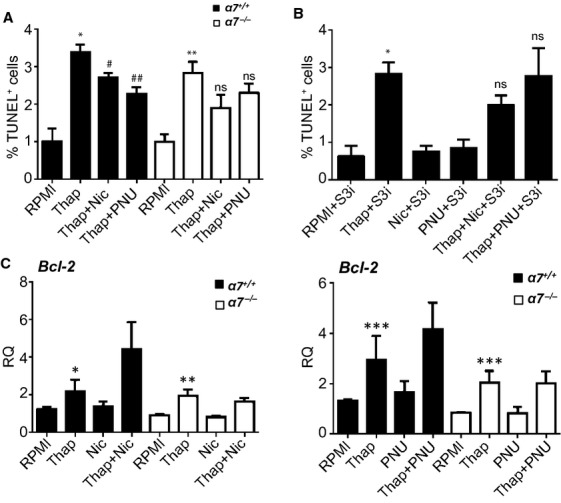
Bone marrow‐derived macrophages (BMDMs) obtained from wild‐type (α7^+/+^) or α7nAChR knockout (α7^−/−^) mice were polarized to the M2 phenotype and incubated for 12 h in: (A) serum‐free RPMI medium alone (RPMI) or containing thapsigargin (1 μmol/L) in the presence or absence of nicotine (10 μmol/L) or PNU‐282987 (1 μmol/L); or (B) as in panel “A”, but in the absence or presence of a STAT3 inhibitor peptide (S3i, 25 μmol/L, 15 min pretreatment) as noted below the graphs. Following treatments, apoptosis was evaluated by the terminal deoxynucleotidyl transferase dUTP nick end labeling (TUNEL) assay. Cells were counted by fluorescence microscopy, and results shown as percentage of TUNEL‐positive nuclei relative to total DAPI‐positive cells. Shown are means ± SEM,* n* = 6. In A): *P = 0.001 and **P = 0.002, compared to RPMI; ^#^*P* = 0.026 and ^##^*P* = 0.005, compared to thapsigargin alone; ns: not statistically different. In (B) *P = 0.0005 compared to RPMI+S3i; ns: not statistically different. (C) BMDMs obtained from wild‐type (α7^+/+^) or α7nAChR knockout (α7^−/−^) mice were polarized to the M2 phenotype, and incubated for 12 h in serum‐free RPMI medium alone (RPMI) or containing thapsigargin (1 μmol/L) in the presence or absence of nicotine (10 μmol/L) or PNU‐282987 (1 μmol/L), as indicated. Following treatments cells were processed for preparation of RNA and cDNA as described in Materials and Methods. Expression levels of Bcl‐2 were measured by qRT‐PCR. Graphs represent data (means ± SEM) of six independent experiments, each performed in triplicates. Gene expression was normalized with GAPDH as an endogenous control. Sequences for primers are provided in Materials and Methods. *P = 0.016, **P = 0.023, ***P = 0.05.

Persistent ER stress is a major mechanism leading to macrophage apoptosis (Tabas 2009). Because we found *α*7nAChR‐dependent activation of survival pathways in polarized BMDMs, we next examined whether *α*7nAChR actually results in modulation of macrophage apoptosis under conditions of ER stress in vitro. M1 and M2 BMDMs were exposed for 12 h to the ER stressor thapsigargin (1 *μ*mol/L) and apoptosis was examined by the TUNEL assay. As shown in Figure 6A, thapsigargin treatment resulted in appearance of a significant number of apoptotic M2 macrophages, regardless of their *α*7nAChR status. Treatment with nicotine or PNU‐282987 alone did not alter the number of apoptotic cells compared to controls (not shown). However, both compounds significantly reduced ER stress‐induced apoptosis in *α*7^+/+^‐M2 (Fig. 6A), but not in *α*7^+/+^‐M1 macrophages (not shown; see footnote^a^). Although a trend toward a protective effect of nicotine remained in *α*7^−/−^‐M2 BMDMs, it did not reach statistical significance (*P* = 0.09); importantly, the proapoptotic effect of thapsigargin was not statistically different between *α*7^+/+^‐ versus *α*7^−/−^‐M2 BMDMs (*P* = 0.17). Remarkably, the protective effect of PNU‐282987 was completely lost in *α*7^−/−^‐M2s. Furthermore, we examined the role of STAT3 in the protective mechanism of nicotine and PNU‐282987 in *α*7^+/+^ M2 BMDMs, using a cell‐permeable STAT3 inhibitor peptide (Cheng et al. 2012). In the presence of the STAT3 inhibitor (25 *μ*mol/L), nicotine treatment consistently showed a trend toward protection against thapsigargin‐induced apoptosis (*P* = 0.07) which, although not reaching statistical significance, suggests that other nAChRs may exert a protective action through STAT3‐independent mechanisms. However, STAT3 inhibition completely abrogated PNU‐282987‐dependent protection from apoptosis (*P* = 0.952 when comparing thapsigargin + PNU‐282987 vs. thapsigargin alone, in the presence of STAT3 inhibitor; Fig. 6B). In the absence of nicotine or PNU‐282987, thapsigargin‐induced apoptosis remained unaffected by inhibition of STAT3 (*P* = 0.20 when comparing thapsigargin‐induced apoptosis in the presence or absence of STAT3 inhibitor). Our results suggest that *α*7nAChR stimulation exerts a selective protection on alternatively activated M2 macrophages from ER stress‐induced apoptosis through a mechanism involving, at least in part, STAT3.

### Activation of *α*7nAChR influences the levels of prosurvival genes during ER stress

Because in M2 BMDMs the observed protective actions of *α*7nAChR against ER stress‐induced apoptosis occur, at least in part, through activation of the STAT3 prosurvival pathway, we speculated that such actions may involve modulation of STAT3‐related survival genes. By means of qRT‐PCR, we examined expression levels of Bcl‐2, a critical survival gene whose expression in macrophages is known to be regulated, to some extent, by STAT3 (Liu et al. 2003). *α*7^+/+^‐ and *α*7^−/−^‐M2 macrophages were exposed to thapsigargin for 12 h, in the presence or absence of nicotine or PNU‐282987 before the isolation of RNA. As shown in Figure 6C, thapsigargin‐induced ER stress resulted in upregulation of Bcl‐2 levels (*P* = 0.037). Exposing macrophages to nicotine or PNU‐282987 under ER stress conditions resulted in a marked trend toward upregulation of Bcl‐2, although it did not reach statistical significance. Interestingly, however, these effects were completely absent in *α*7^−/−^ M2 BMDMs.

## Discussion

The present study examines for the first time a potential role of *α*7nAChR in ER stress‐induced apoptosis in macrophages. We used BMDMs from wild‐type and *α*7nAChR^−/−^ mice to specifically examine the impact of *α*7nAChR deficiency on typical survival mechanisms, apoptosis, and expression of prosurvival genes. Most importantly, these studies were performed on macrophages polarized to the M1 and M2 phenotypes, which are likely to better recapitulate in vitro, the properties and characteristics of macrophage populations found in vivo in the setting of inflammation than those of nonpolarized macrophages. Two salient findings were made in the course of these studies. First, activation of *α*7nAChR resulted in selective activation of the prosurvival JAK2/STAT3 axis in M2 macrophages, but not in M1 cells. Second, *α*7nAChR activation markedly reduced the susceptibility of M2 macrophages to ER stress‐induced apoptosis, an effect that occurred, at least in part, in a STAT3‐dependent manner.

In both M1 and M2 macrophages, we found that typical macrophage survival signaling pathways such as ERK1/2, p38MAPK, and STAT3 were markedly activated by nicotine, a general nAChR agonist. Notably, activation of ERK1/2 and p38MAPK were not affected by pretreating cells with *α*‐bungarotoxin or by *α*7nAChR deficiency, indicating that *α*7nAChR does not play a major role in regulation of these survival routes and that other types of nAChRs might instead be involved. In fact, besides *α*7, PCR studies on cDNA prepared from BMDMs showed expression of the *α*2, 3 and 6 subunits of the nAChRs, but not of *α*1 or *α*9 (not shown). An important derivation from this expression profile is that although *α*‐bungarotoxin is an antagonist of *α*1, *α*7, and *α*9nAChRs (Bray et al. 2005; Albuquerque et al. 2009), in our experimental system its effects can be attributed mostly to its actions on *α*7. Contrarily to what we observed for ERK1/2 and p38MAPK, nicotine‐dependent activation of STAT3 was partially reduced by *α*‐bungarotoxin and by *α*7nAChR deficiency in both M1 and M2 macrophages, suggesting that the *α*7 receptor is required, at least in part, for nicotinic‐dependent activation of STAT3. Notably, activation of STAT3 by nicotine and by the *α*7nAChR selective agonist PNU‐282987 was much more robust in M2 than M1 macrophages. It is plausible to speculate that differences in the signaling environment in each of these macrophage types support a more efficient operation of the *α*7nAChR/JAK2/STAT3 axis in M2 than in M1 cells. In nonpolarized macrophages as well as in cell types other than macrophages nicotine‐dependent activation of STAT3 occurs through a JAK2/STAT3 axis (Marrero and Bencherif 2009; Marrero et al. 2011). Using the JAK2 inhibitor AG490, we here showed that in M1 and M2 macrophages nAChR‐dependent activation of STAT3 also requires JAK2, supporting the notion that similar to peritoneal macrophages (Marrero and Bencherif 2009), an *α*7nAChR/JAK2/STAT3 axis also operates in bone marrow‐derived polarized macrophages, indicating that *α*7nAChR‐dependent modulation of JAK2/STAT3 may represent a general mechanism of nicotinic cholinergic receptor regulation of survival in macrophages regardless of their differentiation status.

Importantly, the present studies show a genuine antiapoptotic effect of *α*7nAChR stimulation when macrophages were exposed to persistent ER stress, a major mechanism leading to macrophage apoptosis in a number of chronic inflammatory diseases, including atherosclerosis. Indeed, by means of an in vitro TUNEL assay, we showed that the prosurvival actions of *α*7nAChR did indeed translate into a reduced susceptibility of M2 macrophages to apoptosis induced by the ER stressor thapsigargin. Notably, this effect occurred, at least in part, in a STAT3‐dependent manner, constituting the first evidence to date linking the *α*7nAChR/JAK2/STAT3 prosurvival axis to reduced macrophage apoptosis.

That in M2 macrophages *α*7nAChR exerts protective, antiapoptotic effects in a STAT3‐dependent manner, is supported by two different yet complementary lines of evidence. First, although nicotine‐dependent STAT3 activation was only partially abrogated by *α*‐bungarotoxin or by lack of expression of *α*7nAChR, nicotine‐dependent protection from ER stress‐induced apoptosis was completely lost in *α*7^−/−^‐M2 macrophages or in wild‐type M2 macrophages treated with a STAT3 inhibitor. These findings also indicate that despite nicotine treatment resulting in significant STAT3 activation in wild‐type and *α*7^−/−^‐M2 macrophages, it is the STAT3 component associated to *α*7nAChR, the one that mostly contributes to protection from ER stress‐induced apoptosis. Second, the *α*7‐selective agonist PNU‐282987 markedly induced STAT3 phosphorylation and reduction in ER stress‐dependent apoptosis in wild‐type, but not in *α*7^−/−^‐M2 macrophages or in wild‐type M2 cells treated with the STAT3 inhibitor.

One of the major mechanisms by which persistent ER stress promotes apoptosis is by induction of C/EBP homologous protein (CHOP), which in turn down regulates expression of antiapoptotic Bcl‐2 family proteins (Oyadomari and Mori 2004; Tsukano et al. 2010). Among others, the Bcl‐2 gene has been shown to be a target of STAT3 (Marrero and Bencherif 2009; Cheng et al. 2012). Our findings show that Bcl‐2 levels were increased in macrophages subjected to thapsigargin‐induced ER stress – likely reflecting compensatory upregulation, as we described previously for macrophages under different proapoptotic conditions (Tano et al. 2012a). Our results show that macrophages exposed to nicotine or PNU‐282987 under ER stress conditions systematically showed a marked trend toward upregulated levels of Bcl‐2. Although this did not reach statistical significance, the fact that this trend was never seen in *α*7^−/−^‐M2 BMDMs is suggestive of the existence of a biological effect of *α*7nAChR on the survival of M2 macrophages. Because thapsigargin causes rapid, robust, and irreversible ER stress, it is possible that this overrides or makes less obvious any protective effects related to *α*7nAChR. Additional studies will be required using submaximal ER stress conditions to define whether Bcl‐2 and/or other STAT3‐related prosurvival genes contribute to the protective actions of *α*7nAChR in M2 macrophages.

A remarkable observation in the present studies is that activation of *α*7nAChR results in a rather selective activation of the JAK2/STAT3 axis in M2 macrophages with a net prosurvival impact on the ability of these cells to cope with sustained ER stress. The question immediately rises as to how activation of *α*7nAChR couples to this survival pathway? The *α*7nAChRs is a homopentamer – (*α*7)_5_nAChRs – which exhibits a typical high permeability to Ca^2+^ (Shen and Yakel 2009). Surprisingly, we did not detect nicotinic‐induced Ca^2+^ influx in BMDMs by means of conventional Fura‐2‐based Ca^2+^ imaging techniques (not shown). In previous study, we showed that both human and murine macrophages are endowed with an efficient Ca^2+^ buffering system (Tano and Vazquez 2011; Tano et al. 2011). Thus, it is possible that Ca^2+^ entering through *α*7nAChRs is rapidly coped by the buffering apparatus therefore going undetectable under conventional imaging conditions. Also, existing evidence indicates that the mode of nAChR‐dependent signaling in immune cells is quite different from the canonical ion channel function typically found in neurons or skeletal muscle, in that the contribution of ion permeation to nAChR signaling in hematopoietic cells seems to be minimal. Rather, agonist‐dependent allosteric transitions seem to modulate the activity of nearby signaling molecules (Hecker et al. 2009; Skok 2009). Additional studies are needed to define whether the role of *α*7nAChR in survival of M2 macrophages relates to channel function or rather reflects a signaling role independent of channeling properties, as described in other cell types (de Jonge and Ulloa 2007).

Over recent years significant progress has been made in our understanding of the role of macrophages, and particularly of macrophage apoptosis, in diseases with a dominant inflammatory component, such as atherosclerosis (Seimon and Tabas 2009; Tano et al. 2012b). Notably, observations made in murine and human lesions point toward a critical role of ER stress‐induced macrophage apoptosis in determining the progression and fate of the atherosclerotic plaque (Scull and Tabas 2011). Macrophages found in atherosclerotic lesions consist of a variety of phenotypes including the polarized M1 and M2 types, among others (Mantovani et al. 2009; Ley et al. 2011) and a number of studies indicate that M2 macrophages are of critical importance particularly in the early disease stages (Khallou‐Laschet et al. 2010a; Lee et al. 2013; Mallavia et al. 2013). In this context, the present findings represent a significant contribution to current efforts in the field aimed at characterizing novel signaling components of mechanisms of macrophage survival and apoptosis that can eventually be exploited as targets for molecular or pharmacological manipulation of macrophage function in the lesion. Ongoing studies in our laboratory will determine whether in vivo and in the inflammatory setting of atherosclerosis the prosurvival actions of *α*7nAChR on macrophages reported here have an actual impact on atherosclerotic lesion development.

## Conflict of Interest

None declared.
